# The Analysis of *In Vivo* Aging in Human Bone Marrow Mesenchymal Stromal Cells Using Colony-Forming Unit-Fibroblast Assay and the CD45^low^CD271^+^ Phenotype

**DOI:** 10.1155/2019/5197983

**Published:** 2019-08-01

**Authors:** Payal Ganguly, Jehan J. El-Jawhari, Agata N. Burska, Frederique Ponchel, Peter V. Giannoudis, Elena A. Jones

**Affiliations:** ^1^Leeds Institute of Rheumatic and Musculoskeletal Medicine, University of Leeds, Leeds, UK; ^2^Department of Clinical Pathology, Faculty of Medicine, Mansoura University, Egypt; ^3^Leeds Musculoskeletal Biomedical Research Centre, Chapel Allerton Hospital, Leeds, UK

## Abstract

Uncultured mesenchymal stromal cells (MSCs) are increasingly used in therapies; however, the effects of donor age on their biological characteristics and gene expression remain unclear. The aim of this study was to investigate age-related changes in bone marrow (BM) MSCs following minimal or no culture manipulation. Iliac crest BM was aspirated from 67 healthy donors (19-89 years old) and directly used for the colony-forming unit-fibroblast (CFU-F) assay or CD45^low^CD271^+^ cell enumeration. The colonies were analysed for colony area and integrated density (ID) when grown in standard MSC media or media supplemented with human serum from young (YS) or old (OS) donors. There was a notable age-related decline in the number of MSCs per millilitre of BM aspirate revealed by the CFU-F assay (*r* = −0.527, *p* < 0.0001) or flow cytometry (*r* = −0.307, *p* = 0.0116). Compared to young donors (19-40 years old), colony IDs were significantly lower in older donors (61-89 years old), particularly for smaller-sized colonies (42% lower, *p* < 0.01). When cultured in media supplemented with OS, young and old donor MSCs formed colonies with lower IDs, by 21%, *p* < 0.0001, and 27%, *p* < 0.05, respectively, indicating the formation of smaller sparser colonies. No significant differences in the expression of selected adipogenic, osteogenic, stromal, and bone remodelling genes as well as CD295, CD146, CD106, and connexin 43 surface molecules were found in sorted CD45^low^CD271^+^ MSCs from young and old donors (*n* = 8 donors each). Altogether, these results show similar trends for age-related decline in BM MSC numbers measured by the CFU-F assay and flow cytometry and reveal age-related effects of human serum on MSC colony formation. No significant differences in selected gene expression in uncultured CD45^low^CD271^+^ MSCs suggest that old donor MSCs may not be inferior in regard to their multipotential functions. Due to large donor-to-donor variation in all donor groups, our data indicate that an individual's chronological age is not a reliable predictor of their MSC number or potency.

## 1. Introduction

Mesenchymal stromal cells (MSCs) are being extensively used as cell therapy or in combination with scaffolds, for the treatment of multiple disorders, owing to their multilineage differentiation and trophic and immunomodulation abilities [1]. Current challenges in this field include a better understanding of MSC tissue diversity, their tissue-specific phenotypes, the impact of aging, and the development of novel protocols for the enhancement of their reparative functions. More recently, strategies based on MSC secretome including extracellular vesicles (EVs) have been proposed as a cell-free alternative [2]. Most of the current treatments utilize large numbers of MSCs and therefore require their large-scale culture expansion. More recent therapies, particularly those for bone and cartilage regeneration, have begun to employ minimally manipulated, autologous MSCs in the form of bone marrow (BM) aspirates or concentrates or loading them on scaffolds [3, 4]. During prolonged cultivation in standard conditions, MSCs undergo an *in vitro* aging process, the highlights of which include telomere erosion [5], gene methylation [6], accumulation of senescent cells [7, 8], and harmful modulation of the membrane glycerophospholipid composition [9]. In combination, this ultimately leads to a gradual loss of potency including MSC homing [10], migration [11, 12], and differentiation capabilities [13] commonly described as MSC “*in vitro* aging.” In contrast, our understanding of how MSC “age” *in vivo*, especially in humans, is limited [13, 14] but remains a key challenge particularly for the translation of minimally manipulated MSC therapies [2].

Most commonly, *in vivo* aging of human MSCs has been investigated indirectly, by comparing the rates of their *in vitro* aging and showing that it happens faster in cultures derived from older individuals compared to cultures from younger donors [15–17]. This indirectly indicates that older donor MSCs might have already “aged” *in vivo* and thus require fewer rounds of *in vitro* cell division prior to reaching senescence. The idea that BM MSCs might “age” *in vivo* is also supported by the fact that several diseases of the musculoskeletal system such as osteoporosis and osteoarthritis are significantly more prevalent in older individuals [18, 19]. To test whether BM MSC numbers decline with age, many studies performed the classical colony-forming unit-fibroblast (CFU-F) assay, which has produced, over the years, somewhat conflicting results: decline [20–22] or no decline [23–25], which can be explained by different protocols used for BM aspiration and processing, as well as variable assay conditions and the differences in colony scoring methods. Even with the assumption that BM MSCs decline with age, it remains unclear whether this possible reduction is due to the preferential loss of the most proliferative cells or is a result of the nonspecific decline in all types of colony-forming progenitors. The data on quantification of BM MSCs using flow cytometry based on the specific MSC surface phenotype also remains limited [26–30] and requires further independent verification between different laboratories.

Similarly, the relationships between donor age and their MSC gene expression, prior to cultivation, remain poorly understood. The literature pertaining to cultured MSCs suggests a potential shift from their osteogenic to adipogenic fate with increasing donor age [13, 31]; however, this has not been confirmed using uncultured MSCs. Finally, several studies utilizing cultured MSCs have proposed certain surface molecules associated with MSC “youthful” or “aged” status, including CD106 [32], CD146 [17], or CD295 [33], but these molecules have not been comprehensively studied on uncultured MSCs in relation to donor age. In this study, we hypothesised that age-related changes in BM MSCs are associated with a preferential loss of the most proliferative clonogenic progenitors and that their gene expression profile reflects a shift from osteogenesis and hematopoietic stromal support activity to adipogenesis. We also hypothesised that MSCs from older individuals possessed higher levels of CD295 expression mirroring previous findings obtained from cultured MSCs [33], as well as the reduced levels of connexin 43, based on our previous gene expression data [34].

To investigate this, we used the classical CFU-F assay as well as flow cytometry/cell sorting for the CD45^low^CD271^+^ cell population, a recognized phenotype of human BM MSCs [26, 28, 29, 35, 36]. Previous independent studies have documented that this cell subset fully fulfils the MSC criteria as stipulated by the International Society for Cell Therapy position statement [37]: the expression of CD73/CD90/CD105 [34, 38, 39], high-level enrichment on colony-forming cells [39, 40], and multipotential differentiation capacity [34, 40]. We used a large cohort of BM donors aged from 19 to 89 years old, who underwent surgeries for orthopaedic trauma or metalwork removal, but were otherwise healthy. The CFU-F assay was performed volumetrically, without additional processing steps that could have led to the loss of MSCs [27], and the colonies were further analysed for colony areas and integrated densities, in order to discern any differences in CFU-F distribution, in relation to donor age. The effects of an “aging environment” on MSC colony formation were further analysed using media supplemented with human serum.

## 2. Methods

### 2.1. Sample Collection and CFU-F Assay

Ethical approval for the collection of samples was obtained from NREC Yorkshire and Humberside National Research Ethics Committee (number 06/Q1206/127). Bone marrow aspirate (BMA, 10 ml) was collected from the iliac crest (IC) of 33 female and 34 male patients with a median age of 49 years (range 19-89) and used immediately upon receipt. The donors were admitted at the Leeds General Infirmary for orthopaedic surgery, and donors with any metabolic diseases, systemic illness, or cancer were excluded. All the aspirations were performed by the same surgeon and under the same conditions for consistency.

BMA was first passed through a sterile 70 *μ*m cell strainer (Falcon, Fisher Scientific, Loughborough, UK) to exclude fat or bone debris. Subsequently, enumeration of MSCs was performed using the CFU-F assay and flow cytometry followed by freezing of the remaining nucleated cells after ammonium chloride lysis of erythroid lineage cells, as described previously [41]. In brief, ammonium chloride was added to BMA in an 8 : 1 ratio and incubated at 37°C for 10 minutes. The cells were then washed three times with phosphate-buffered saline (PBS, Life Technologies, Paisley, UK) then frozen.

For the CFU-F assay, 80 *μ*l of BMA was added directly into each of the duplicate 60 mm petri dishes (Corning, New York, USA) containing 4 ml of StemMACS MSC expansion media (SM) (Miltenyi Biotec, Bisley, UK) with 1% Penicillin-Streptomycin (P/S, Thermo Scientific, Loughborough, UK). Following the mixing of BMA and media, dishes were placed in the incubator in 37°C and 5% CO_2_ and maintained in culture for two weeks. After 48 hours, the dishes were gently washed with PBS and 4 ml of fresh SM was added. Thereafter, half media change was performed twice a week, and on the 14^th^ day, the resulting colonies were fixed with 3.7% formaldehyde (Fisher Scientific, Loughborough, UK) stained with 1% *w*/*v* methylene blue (Sigma, Dorset, UK) and scanned. A colony was defined as consisting of at least 50 cells [29]. The colonies were manually counted and CFU-F/ml was calculated according to the formula (1000 *μ*l × average number of colonies from 2 dishes)/80 *μ*l, and the results were plotted against donor's age for correlation. Additionally, the analysis was performed between three different age groups denoted as young (19-40 years old), intermediate age (41-60 years old), and old (61-89 years old), as defined in previous studies [16, 20, 21].

### 2.2. Analysis of Colony Area and Integrated Density

Colony sizes and densities have been previously reported to reflect individual MSC's proliferative capacities [42, 43]. To investigate this in relation to donor age, CFU-F dishes with similar numbers of colonies (average 40 colonies per dish, range 25-60) from the young and old age groups (*n* = 7 donors each) were analysed for colony area and integrated density (ID). The images of scanned dishes were converted to “grey scale” to be recognized by ImageJ software, which generated an outline for each colony. In order to avoid the influence of the background disturbances and to minimize software error (for example, by counting two overlapping colonies as one), the colonies on the edges of the dish were given an outline manually. The values for colony area and ID were generated simultaneously, and IDs were calculated as [area^∗^mean grey value]. Once the data for colony area and ID were recorded, the data were compared between the young and old donor groups. For the subsequent analysis, colonies were segregated as small or large based on the median value obtained from all colonies (below and above the median, respectively), and their IDs were analysed separately and also in relation to donor age.

### 2.3. MSC Colony Formation in Media Containing Human Serum

To investigate the proliferative capacity of MSCs that would mirror conditions *in vivo*, human serum was prepared and used as the *in vivo* microenvironment. The aim of these experiments was to investigate how young and old donor MSCs responded to young and old donor human serum in terms of their colony numbers, areas, and IDs. To prepare human serum, 30 ml of blood was collected from 4 healthy old (59-62 years old) and 4 healthy young (23-31 years old) donors and serum was prepared by spinning the blood at 2000g for 15 minutes. Subsequently, 4 sera were pooled together to generate stocks of old donor serum (OS) and young donor serum (YS) separately, which were then aliquoted and frozen at -20°. Media containing either 10% YS or 10% OS were prepared fresh using DMEM as the basal media (Life Technologies, Paisley, UK) and supplemented with 1% P/S.

Frozen nucleated cells (from donors with at least 10^7^ frozen cells) were defrosted at 37°C in thawing media (DMEM/10% FCS) (Thermo Scientific, Loughborough, UK) containing 1% P/S and 20 units/ml DNAse (Sigma, Dorset, UK). Following centrifugation, the cells were equally divided into nine 60 mm dishes containing 4 ml SM for an initial 48-hour attachment period. Subsequently, the media were removed, the adherent cells washed with PBS, and complete media changes were performed as follows: 3 dishes were supplemented with 4 ml DMEM/10% YS, 3 dishes with 4 ml of DMEM/10% OS, and 3 final dishes with 4 ml SM (control). The dishes were then placed at 37°C and 5% CO_2_ for 2 weeks, and half media changes were performed using the respective media twice a week. On day 14, all media were aspirated, and for each condition, two dishes were stained with methylene blue to visualise the colonies, while the adherent cells from the third dish were detached using trypsin (Life Technologies, Paisley, UK) and counted. The stained dishes were analysed for colony area and ID, as described in the previous section.

### 2.4. Enumeration of BM MSCs Using CD45^low^CD271^+^ Phenotype

MSC enumeration by flow cytometry was performed on the same cohort of donors in order to directly compare the data with the results of the CFU-F assay. For this, 100 *μ*l of BMA was placed in a FACS tube (Falcon) containing 20 *μ*l CD271-allophycocyanine (APC) (Miltenyi Biotec) and 20 *μ*l CD45-phycoerythrin- (PE-) cyanine dye 7 (Cy7) (BD Pharmingen, Oxford, UK) for 15 minutes at room temperature (RT); 10 *μ*l of 7-Aminoactinomycin-D (7-AAD, BD Pharmingen) was added simultaneously in order to distinguish between live and dead cells. After staining, erythroid lineage cells were lysed using ammonium chloride (Sigma, Dorset, UK) at 37°C for 10 minutes. Finally, 50 *μ*l of CountBright absolute counting beads (Life Technologies) was added at RT after a quick vortex for 5-7 seconds, as previously described [27]. The data were acquired using the Becton Dickinson (BD) LSRII 4 laser flow cytometer. Unstained and single antibody-stained controls were used to optimize the cytometer voltage settings and spectral compensation, and isotype controls (IgG1-APC and IgG1-PE-Cy7, BD Pharmingen) were used to gate for MSCs. The analysis of the flow cytometry data was performed using the FACS Diva software (BD Biosciences, Oxford, UK), as previously described [27].

### 2.5. Cell Sorting and Surface Marker Expression Analysis

Frozen nucleated cells were defrosted (*n* = 8 donors from each young and old groups), and MSCs were enriched using Anti-Fibroblast beads (Miltenyi Biotec), as previously described [34]. Enriched cells were treated with Fc block (Miltenyi Biotec) for 10 minutes to prevent nonspecific antibody binding and then stained with the following antibodies for 15 minutes: 20 *μ*l CD45 V450, 20 *μ*l CD271 PE Vio770, 20 *μ*l CD106 PE, and 10 *μ*l CD295 Alexa Fluor 647 (panel 1); or 20 *μ*l CD45 V450, 20 *μ*l CD271 PE Vio770, 20 *μ*l CD146 PE (all from BD Pharmingen, Oxford, UK), and 10 *μ*l Cx43 APC (R&D Systems, Minneapolis, USA) (panel 2). Isotype controls for each antibody were used to control for nonspecific antibody binding. After staining, the cells were washed in FACS buffer and passed through the filtered-cap flow tubes (Falcon) to remove any cellular clumps. Finally, 10 *μ*l of 7-AAD was added to all the tubes prior to cell sorting using BD Influx cell sorter.

Instrument settings and spectral compensations were performed using unstained cells, compensation beads, and isotype controls. Cells were sorted into 2 tubes to collect the following populations: CD45^low^CD271^+^ (MSCs) and CD45^high^CD271^−^ (hematopoietic lineage cells or HLCs), as previously described [34]. Sorted cells were directly collected into 1.5 ml Eppendorf tubes containing 350 *μ*l guanidinium-based lysis buffer (Buffer RL, Norgen, Thorold, Canada), and lysates were frozen at -80° for subsequent RNA extraction and qPCR. Surface marker expression on gated CD45^low^CD271^+^ MSCs and CD45^high^CD271^−^ (HLCs) was analysed using FlowJo Software (BD, version 10).

### 2.6. Gene Expression

RNA was extracted from cell lysates (obtained from cell sorts) using Single Cell RNA Purification Kit (Norgen Biotek, Canada) following manufacturer instructions with genomic DNA removal using DNAse I from the same company. RNA was eluted in 15 *μ*l of elution solution, evaluated for its concentration and integrity using Nanodrop and stored in -80°C until further use. 2 *μ*l of RNA was reversely transcribed (RT) to cDNA using RT reagent (Fluidigm, California, USA), and the reaction was carried out in thermal cycler (Applied Biosystems, California, USA) under the following conditions: 5 min at 25°C, 30 min at 42°C, 5 min at 85°C, and then held at 4°C. cDNA was stored in -20°C until analysis. For preamplification (PA), a mixture of 12 TaqMan Gene Expression assays (Applied Biosystems) at a final concentration of 0.2x for each assay (Supplementary Table 1) was prepared with the PA reagent (Fluidigm) and 1.25 *μ*l of cDNA. All samples were preamplified in the thermocycler using 18 cycles polymerised chain reaction (PCR) following the settings 2 min at 95°C, 15 sec at 95°C, 4 min at 60°C, and then held at 4°C. After preamplification PCR cDNA was diluted 1 : 5 with Tris and Ethylenediaminetetraacetic acid (TE) buffer and was stored at −20°C before processing on the BioMark HD. Quantitative PCR (qPCR) was performed using Fluidigm FLEXSIX™ integrated fluid circuits (IFC) on BioMark HD. For qPCR, 2 *μ*l of each TaqMan assay was mixed with 2 *μ*l Gene Expression Assay Loading Reagent (Fluidigm). Then, 1.8 *μ*l of diluted cDNA was mixed with 2 *μ*l 2× TaqMan Universal Mastermix (Thermo Fisher) and 0.2 *μ*l Gene Expression Sample Loading Reagent (Fluidigm). Next, 3 *μ*l of each sample and assay was loaded into individual sample and assay inlets on the IFC. Samples and assays were then loaded into the reaction chambers of the FLEXSIX using the IFC Controller HX (Fluidigm) and then transferred to the BioMark HD for qPCR (95°C for 10 min; 40 cycles of 95°C for 15 seconds, and 60°C for 60 sec). The Ct values for genes of interest were normalised to the endogenous control HPRT-1 using the formula [ΔCt = Ct_target gene_ − Ct_housekeeping gene_], and relative expression was calculated as 2^-*Δ*Ct^ and used for statistical analysis.

### 2.7. Statistics

Statistical analysis and graphics were performed using GraphPad Prism software (version 7.0a). The normal distribution of the data was assessed using the Shapiro-Wilk and Kolmogorov-Smirnov tests for normality. As no data were found to be normally distributed, Mann-Whitney test and Kruskal-Wallis test with Dunn's correction for multiple comparisons were used to compare two and three groups, respectively. Spearman test was used to analyse correlations. The results were considered significant at *p* value of <0.05.

## 3. Results

### 3.1. MSC Enumeration and Colony Characterisation Using Standard CFU-F Assay

**Figure 1:**
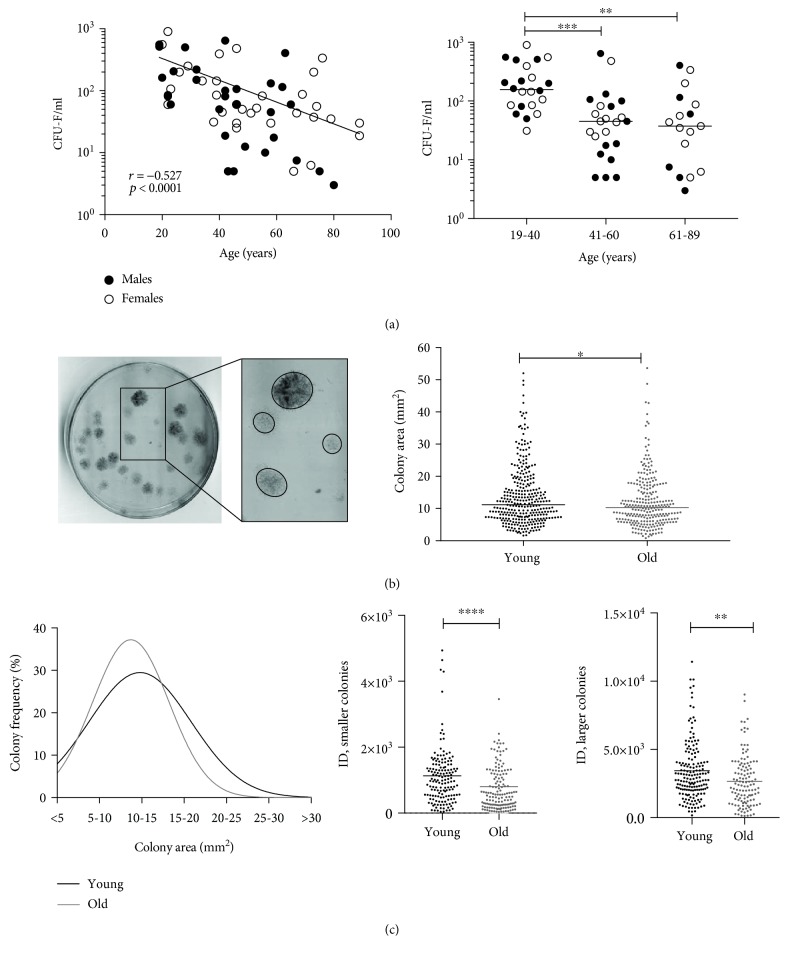
Age-related decline in number and proliferative capacity of BM MSCs. (a) Correlation between CFU-Fs/ml of BM aspirate with donor age across complete age range (left, *n* = 62 donors, 19-89 years old) and between three age groups (right); black lines indicate the line of the best fit (left) and median values for each group (right). (b) Representative colony dish showing the presence of large and small colonies (left) and comparison of colony areas in all colonies in 7 young donors (19-37 years old, 321 colonies) and 7 old donors (61-76 years old, 266 colonies) (right). Each dot represents a single colony; black lines represent median values. (c) Frequency distribution of colony areas for colonies from the same 7 young (black curve) and 7 old donors (grey curve) (left) and comparison of integrated densities (IDs) of smaller colonies (left) and larger colonies (right) in the young and old donor groups. ^∗^ *p* < 0.05, ^∗∗^ *p* < 0.01, ^∗∗∗^ *p* < 0.001, and ^∗∗∗∗^ *p* < 0.0001.

BM MSCs from 62 donors (*n* = 33 females, *n* = 29 males) were first quantified using the standard CFU-F assay following a 14-day culture in SM media, and a significant age-related decline in the number of colonies was found (*r* = −0.527, *p* < 0.0001) (Figure 1(a), left panel). When the whole cohort was divided into three age groups, young (19-40), intermediate age (41-60), and old (61-89 years old), a significant 4.2-fold decline was observed in the number of MSCs in the old group (median 37 CFU-F/ml) compared to the young group (median 156 CFU-F/ml) as well as a 3.5-fold decline in the intermediate age group (median 45 CFU-F/ml) compared to the young group (Figure 1(a), right panel). There was no statistically significant difference in the average number of colonies between donors of the intermediate age group and the old group for all donors, as well as when separating male and female cohorts (Figure 1(a)). Notably, high donor-to-donor variation was observed in all donor groups, and some old donors had CFU-F numbers within the range of the young donor group (Figure 1(a), right panel).

To investigate whether a decline in colony number in the old donor group was associated with a specific loss of the most proliferative progenitors, colony area and ID analysis was performed on the CFU-F dishes from 7 young and 7 old donors containing a minimum of 25 colonies per dish. A total of 587 colonies (266 and 321 colonies from the old and young groups, respectively) were analysed using ImageJ and revealed CFU-F colonies of vastly different sizes in both age groups (Figure 1(b), left panel). However, colonies from the old group were on average 10% smaller than colonies from young donors (Figure 1(b), right panel). The frequency distribution of colony areas was next performed using GraphPad Prism software by generating a nonlinear fit line based on modal values of predefined bin widths of 5 mm^2^. This analysis revealed unimodal colony area distributions in both young and old cohorts; however, a prominent shift towards smaller colonies was observed in the old group (Figure 1(c), left panel).

Colonies with the smallest sizes and lowest densities have previously been suggested to derive from the least proliferative, presenescent progenitor cells [43]. As colony's ID is a combined measure of colony's size and its density, the largest ID values indicate large, dense colonies (i.e., derived from the most proliferative MSCs), while the smallest ID values correspond to small, sparse colonies (derived from the least proliferative MSCs) [42]. To further investigate these colony types in relation to donor age, the median area of all 587 colonies (10.64 mm^2^) was set as the cutoff point to separate smaller and larger colonies, and their IDs were compared between the donor groups (Figure 1(c), right panel). For both, smaller and larger colonies, their IDs were significantly lower in the old donors (42% and 18% lower, respectively); however, the largest decline in IDs was observed for smaller colonies (Figure 1(c), right panel).

Altogether, CFU-F data revealed a gradual age-related decline in the number of colony-forming MSCs, as well as colony areas and IDs (particularly for smaller colonies), indicating increased proportions of less proliferative MSCs in old donors BM.

### 3.2. MSC Enumeration and Colony Characterisation in Conditions Containing Human Serum

**Figure 2:**
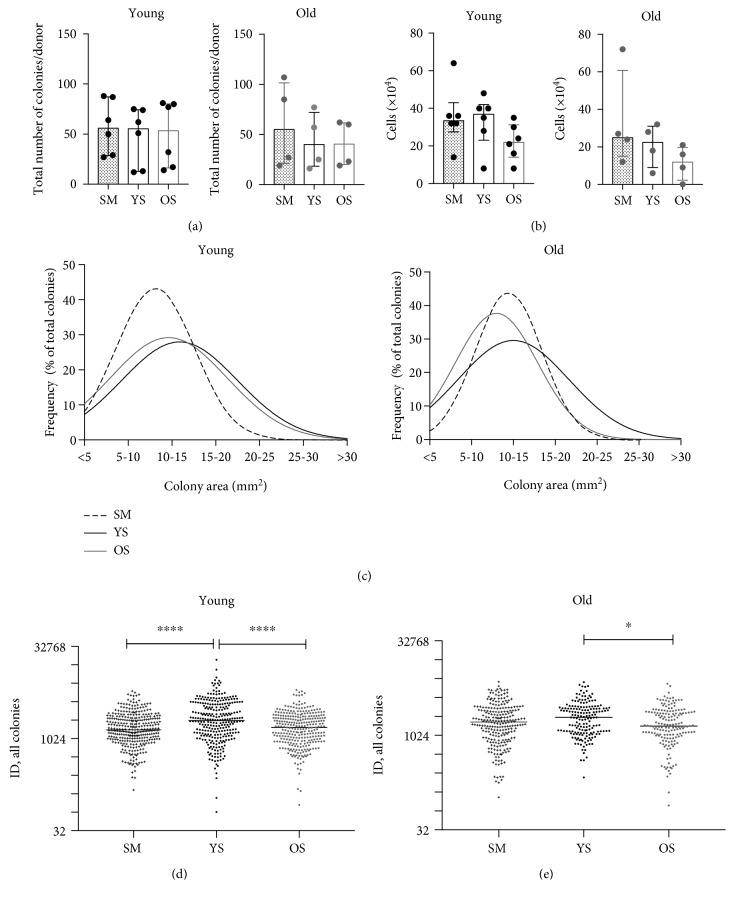
MSC colony formation in media containing human serum. (a) Total number of colonies from 6 young (19-37 years old, left) and 4 old donors (65-79 years old, right), grown in StemMACS (SM) or media containing young donor serum (YS) or old donor serum (OS). (b) Cell counts from cells grown in each of the serum conditions in the same 6 young (left) and 4 old (right) donor groups. Bars indicate median values and whiskers represent interquartile ranges. (c) Frequency distribution of colony areas for all colonies from the same 6 young (left) and 4 old (right) donors grown in SM, YS, or OS conditions. (d) Comparison of IDs of all colonies from the same 6 young donors grown in SM (345 colonies), YS (286 colonies), and OS (301 colonies) conditions. (e) Comparison of IDs of all colonies from the same 4 old donors grown in SM (241 colonies), YS (175 colonies), and OS (170 colonies) conditions. Data in (d) and (e) is presented on log_2_ scale. Black lines represent median values. ^∗^ *p* < 0.05 and ^∗∗∗∗^ *p* < 0.0001.

Although the CFU-F assay conducted in media preselected for enhanced MSC growth still represents the “gold standard” for MSC enumeration [44], cell cultivation is notably performed in conditions drastically different to which they are normally exposed *in vivo*. To mimic old donor MSC exposure to their more natural environment, CFU-F assays were next conducted in media containing human serum. The assays were performed using frozen/thawed nucleated cells from the young and old donor groups, which were all initially plated in SM to allow for equal attachment and 48 hours later transferred to media containing pooled human serum from either young (YS) or old (OS) donors, or SM as controls. A total of 1517 colonies were analysed in these experiments, 932 and 586 from the young and old donor groups, respectively. In addition to staining dishes and analysing colony data, the cells from the third dish were trypsinised and counted to obtain data on the produced total cell yields (Figure 2).

The numbers of colonies grown in media supplemented with human serum were similar to SM conditions and showed no significant difference between YS and OS conditions, in both young and old donors (Figure 2(a)). However, the analysis of cell counts obtained from the third dish revealed a trend for a decline in the number of cells obtained from both young and old donor groups when cultured in OS as compared to YS (Figure 2(b)) indicating the reduced proliferation of MSCs in OS. Cell counts obtained from dishes grown in YS conditions were similar to control SM conditions (Figure 2(b)). To dissect this further, colony area and ID analysis was performed on all colonies. In the young donor group, the frequency distributions of colony areas were quite similar for OS or YS culture conditions (Figure 2(c), left panel), with the most frequent colonies being larger in area than colonies grown in SM (dashed line). However, in the old donor group, OS conditions have led to shift towards smaller colonies, which became even smaller than SM colonies (Figure 2(c), right panel). Of note, colony distributions in SM from frozen cells (Figure 2(c)) were different to colony distributions from fresh cells (Figure 1(c)) possibly due to the fact that frozen/thawed cells required some time to recover from the frozen state before they began proliferating and forming a colony.

In the young donors' group, the ID of colonies grown in YS was significantly 21% higher (*p* < 0.0001) compared to OS conditions and significantly 31% higher (*p* < 0.0001) compared to SM conditions (Figure 2(d)). In the old donors' group, the ID of colonies grown in YS was similarly, significantly 27% higher (*p* < 0.05) compared to OS conditions, but differences in colony IDs in comparison to SM were not statistically significant (Figure 2(e)).

### 3.3. MSC Enumeration and Gene Expression Using CD45^low^CD271^+^ Phenotype

**Figure 3:**
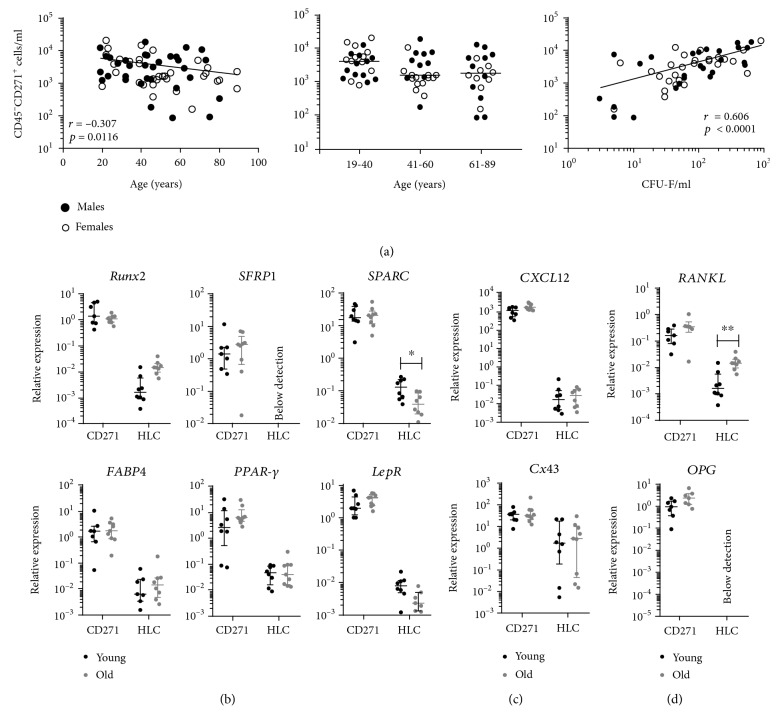
BM MSC enumeration and gene expression using CD45^low^CD271^+^ phenotype. (a) Correlation between MSCs identified as CD45^low^CD271^+^ cells with donor age across complete age range (total 67 donors, left), between three age groups (centre), and between CFU-F/ml and donor-matched CD45^low^CD271^+^ cells/ml (right). Black lines across data sets indicate line of best fit (left and right) and median values for each group (centre). (b) Expression of transcripts associated with osteogenic (top) and adipogenic differentiation (bottom). (c) Expression of transcripts associated with intercellular communication. (d) Expression of transcripts associated with bone remodelling. Black and grey lines indicate median values with interquartile range in young and old donors, respectively. ^∗^ *p* < 0.05, ^∗∗^ *p* < 0.01; *n* = 8 young donors (19-38 years old) and 8 old donors (61-89 years old). CD271: CD45^low^CD271^+^ MSCs; HLC: CD45^high^CD271^−^ hematopoietic lineage cells.

We and the others have previously reported that all CFU-Fs in human BM are present in a rare cell population characterised by the CD45^low^CD271^+^ phenotype [26, 40]. BM MSCs from the same cohort of donors were next enumerated using CD45^low^CD271^+^ phenotype (Figure 3(a)). Similar to our CFU-F data, there was an age-related decline in the numbers of CD45^low^CD271^+^ cells (*r* = −0.307, *p* = 0.0116); however, the slope of the decline was much shallower (Figure 3(a), left panel). When the whole cohort was divided into three age groups, young, intermediate age, and old, there was a 2.2-fold decline in the number of CD45^low^CD271^+^ cells in old donors (median 1825 cells/ml) and a 2.6-fold decline in donors from intermediate age (median 1587 cells/ml) compared to young donors (median 4103 cells/ml) (Figure 3(a), middle panel), a pattern similar to CFU-Fs, but failing to reach statistical significance. As expected [27, 29], there was a strong positive correlation between CFU-F and CD45^low^CD271^+^ cell counts (*r* = 0.606, *p* < 0.0001) (Figure 3(a), right panel). Based on the respective median values, the CFU-F/CD45^low^CD271^+^ cell ratios were 1 in 26 in young donors, 1 in 35 in donors of intermediate age, and 1 in 48 in old donors suggesting an age-related decline in the colony-forming ability of CD45^low^CD271^+^ cells.

In terms of their gene expression, there was no significant difference in the expression of selected transcripts related to osteogenic or adipogenic differentiation (Figure 3(b)). We also found no differences in transcripts associated with cellular interaction (Figure 3(c)) or bone remodelling (Figure 3(d)) in CD45^low^CD271^+^ cells. However, the trend for increased *RANKL* and *OPG* was noted in CD45^low^CD271^+^ cells from old donors, as well as significantly higher *RANKL* in old donor HLCs (Figure 3(d)). Transcripts of *OPG* and *SFRP-1* could not be detected in HLCs while the remaining molecules were significantly higher in CD45^low^CD271^+^ cells compared to HLCs (Supplementary Table 2) confirming their MSC specific identity.

### 3.4. Leptin Receptor, Cx43/Connexin 43, and Other Surface Marker Expression in CD45^low^CD271^+^ Cells

**Figure 4:**
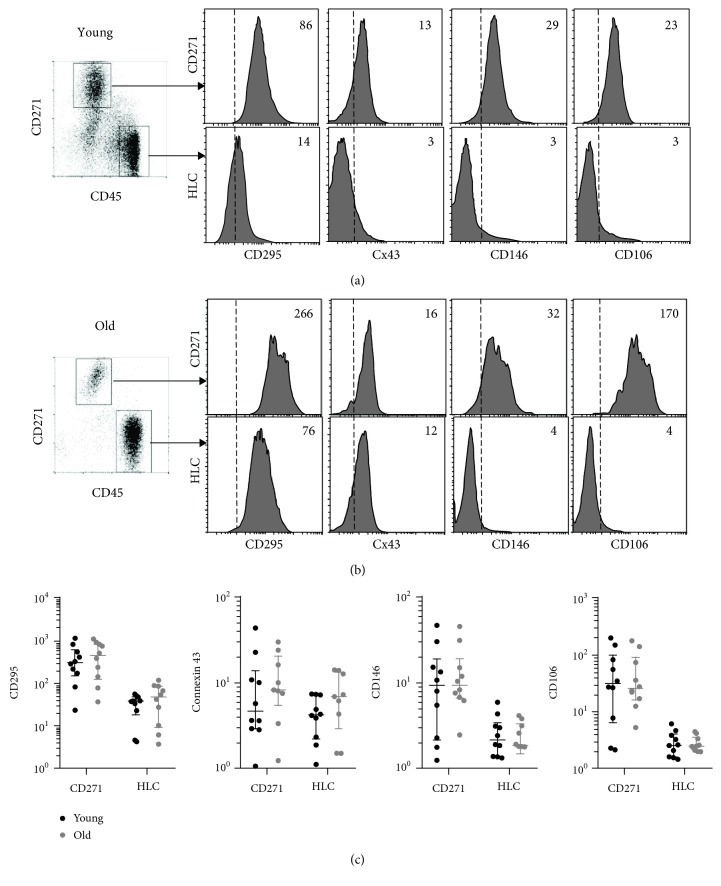
Surface marker expression in CD45^low^CD271^+^ cells. (a) Gated populations and marker histograms from representative young (19 years old) donor showing MSCs (top panel) and HLCs (bottom panel) with median fluorescence intensity (MFI) indicated on the right top corner. (b) Gated populations and marker histograms from representative old (67 years old) donor showing MSCs (top panel) and HLCs (bottom panel) with MFI indicated on the right top corner. (c) Comparison of expression of selected surface markers in 8 young (19-38 years old, black) and 8 old (61-89 years old, grey) donor MSCs and HLCs with lines indicating median values with interquartile ranges for each group. CD271: CD45^low^CD271^+^ MSCs; HLC: CD45^high^CD271^−^ hematopoietic lineage cells.

The leptin receptor/CD295 and Cx43/connexin 43 expression on uncultured human BM MSCs was next analysed using flow cytometry. Also, the expression of two other markers CD146 and CD106 previously linked to MSC “youthful” or “aged” [21, 34] status was assessed simultaneously. Population gating was performed on CD45^low^CD271^+^ cells (MSCs) and control CD45^high^CD271^−^ cells (HLCs) (Figures 4(a) and 4(b), left panels). The expression of CD295, CD146, and CD106 was significantly higher in MSCs compared to HLCs (Supplementary Table 2). Representative histograms are shown in Figures 4(a) and 4(b). However, there were no age-related differences in the level of expression of any of these molecules in MSCs as well as HLCs (Figure 4(c)).

## 4. Discussion

There is a significant body of literature investigating the phenomenon on *in vitro* aging of human BM MSCs. In accumulation, it describes the gradual loss of proliferation, telomere erosion, and the modulation of MSC gene and protein expression, as a consequence of accrued population doublings (commonly 20-40) [14, 44, 45]. On the other hand, the evidence of human BM MSC “aging” *in vivo* remains scarce. It is commonly extrapolated indirectly from histological, serological, and functional studies of unfractionated bone or BM samples from young and aged donors and suggests “differentiation reprogramming of BMSC toward adipocyte instead of osteoblastic fate,” reviewed in [31]. However, this has not been thoroughly investigated using purified uncultured BM MSCs.

In this work, we used minimal MSC culture in the CFU-F assay format to study proliferation of single adherent colony-forming cells in relation to donor age. Sample collection and processing were tightly controlled for BM aspiration and processing, and a large cohort of individuals was recruited. Consistent with published studies [5, 15, 20–22], we observed a gradual decline in CFU-Fs with increasing donor age; however, when the cohort was split into three age groups, spanning two decades each, the bigger reduction in CFU-F numbers was noted in the intermediate age group, compared to the young age group, and not in the old group compared to the intermediate age group. This was similar to the previously reported data in Siegel et al.'s study [46] and could be related to the highest bone turnover rates and hence a demand for MSCs in individuals in their midtwenties (at their peak bone mass), after which this demand declines gradually [47]. In agreement with this idea, previous studies have documented significantly higher BM MSCs in children and adolescents compared to adults and the elderly [20, 21, 48].

We next focussed on measuring colonies' areas and integrated densities in order to potentially identify a particular colony type [42] that could be specifically affected in older age. Colony size distributions revealed no distinct segregation into “large” and “small” colonies; instead, the colony distributions in both younger and older donor groups were unimodal. Nevertheless, compared to young donors, the integrated densities of colonies from old donors were significantly lower, suggesting their reduced proliferative abilities. From these results, it could be concluded that age-related changes in human BM MSCs are associated with a general reduction in proliferative capacities of all colony-forming cells, rather a specific loss of the most proliferative progenitor cell type.

We were mindful that these findings could simply reflect human MSC responses to specific CFU-F assay conditions, in which culture media/serum are selected by the manufacturers or researchers to specifically enhance their *in vitro* proliferation and expansion potentials. In order to validate these findings using more “natural” culture conditions, we performed similar experiments in media supplemented with human serum. The effects of human serum on MSCs have been studied before [49, 50], but not at the single colony level. In this study, we found that old donor serum had a negative impact on colonies grown from both young and old donors (they became smaller and less dense), possibly by increasing oxidative stress levels in cells, as shown in a recent rat model study [51], or via higher levels of proinflammatory cytokines that are known to inhibit MSC proliferation [52]. Correlating pro- and anti-inflammatory cytokine levels in serum from young and old donors with their BM MSC number and colony IDs or performing serum supplementation experiments with the addition of antioxidants such as N-acetyl-cysteine or superoxide dismutase may help to further address the mechanisms of MSC proliferation inhibition. Conversely, the integrated density of colonies from the young donor group could be improved by cultivation in media containing young donor serum as compared to old donor serum (by 21%, *p* < 0.0001) or SM (by 31%, *p* < 0.0001). A similar trend was observed for the old donor group, but a significant difference (27%, *p* < 0.05) was only observed between YS and OS. The limited responsiveness of old donor MSCs could be due to reduced receptor levels for proliferation-promoting growth factors, such as platelet-derived growth factors [53] on their MSC surface. Altogether, these data indicated that the type of serum is an important factor to consider in MSC aging studies, even if MSCs are only cultured for a limited period of time. Nevertheless, colony numbers grown in human serum were not markedly different to those obtained in the optimized expansion media confirming the reliability of MSC enumeration by colony scoring in FCS-containing media. Our data also indicated that culturing old donor MSCs in media containing young donor serum could marginally “rejuvenate” their proliferation ability. Different strategies including growing old donor MSCs on a young donor extracellular matrix [54, 55] or with the addition of young donor platelet lysate [56], as shown in previous studies, may produce more substantial outcomes.

Since even minimal MSC expansion in different human sera has led to measurable differences in colony densities, we next focussed on analysing *in vivo* MSC aging without resorting to any expansion *in vitro*. For this, we chose to quantify CD45^low^CD271^+^ cells, a recognized phenotype of BM MSCs, which has been validated in previous independent studies [26, 28, 29, 36], as well as of MSCs resident in the adipose tissue [28, 57]. We compared the flow cytometry results with our CFU-F findings, which were performed using the same cohort of donors. While the results from the two assays significantly correlated with each other, the age-related decline in CD45^low^CD271^+^ cell population was notably less pronounced than for CFU-Fs. This could be explained by the fact that only a proportion of CD45^low^CD271^+^ cells can generate a colony in standard conditions (variable from 1 in 17 [27] to 1 in 40 [40]), which in this study was approximated to 1 in 26 or 1 in 48 in the young and old groups, respectively. These results indicated an age-related decline in MSC proliferation, confirming our colony number and integrated density data. A further study investigating the proportions of senescent cells in rare sorted CD45^low^CD271^+^ cells from young and old donors, using emerging methodologies [58], will help to clarify this point.

From MSC enumeration experiments, we next proceeded to measuring their osteo- and adipogenesis-related gene expression using purified CD45^low^CD271^+^ cells. The data generated from long-term cultured MSCs (representing their “*in vitro* aging”) remain controversial. For example, some studies including ours have previously shown increased or unchanged levels of osteogenesis-related molecules, in “*in vitro* aged” MSCs, paralleled with a decrease in adipogenesis-related molecules [59, 60], in line with functional measurements of their osteo- and adipogenic potentials as functions of passages [7]. On the other hand, *in vivo* observations of diminished trabecular bone volume [61] and increased BM adiposity with increasing age [31] suggested otherwise. In this study, we found no major differences in adipogenesis-related transcript levels in CD45^low^CD271^+^ cells from younger and older donors. The transcripts for osteogenesis-related molecules from CD45^low^CD271^+^ cells also showed no age-related differences. In a separate study using a smaller cohort of donors (*n* = 6, age range 24-62 years), we performed CFU-O (osteoblast) colony assays in osteogenic media and no particular differences in the proportions of alkaline phosphatase-positive colonies were observed in relation to donor age. These preliminary experiments support our current findings on the similar basal levels of osteogenic gene expression in MSCs from old and young donors and require further validation using larger donor cohorts. Interestingly, the use of unfractionated bone marrow to study the expression of mesenchymal lineage-defining transcripts revealed no significant age relationships as well [62].

No differences in the expression were similarly found for selected chemokine *CXCL12* as well as *Cx43/*connexin 43 known to be involved in MSC communication with other cells including hematopoietic stem cells [63]. This was unexpected as higher *Cx43* transcript levels have been previously found in paediatric CD45^low^CD271^+^ cells compared to their adult counterparts [34] and in young rat BM MSCs compared to old rat MSCs [64]. Finally, interesting trends were noted for the expression of genes involved in bone remodelling, with both *RANKL/*receptor activator of nuclear factor kappa-B ligand and its decoy receptor *OPG/*osteoprotegerin showing higher expression levels in the older donor group. Interestingly, recent experiments with mouse MSCs have indicated a link between increased expression of *RANKL* and age-related marrow adipogenesis [65].

Leptin receptor (LepR) is a well-known MSC-specific molecule in mice [66, 67] and is involved in controlling marrow bone-fat balance in humans [68, 69]. We measured *LepR* levels in CD45^low^CD271^+^ cells, both at the gene expression and protein levels and found its significantly higher expression in MSCs compared to control HLCs, but no differences in between younger and older donor groups. In contrast, LepR upregulation was described in *in vitro* aged MSCs and linked with an increase is a subpopulation of dying cells [33]. Finally, we used flow cytometry to investigate the expression of two other molecules (CD146 and CD106) previously linked to *in vitro* MSC aging, on uncultured CD45^low^CD271^+^ BM cells. CD146 expression has been previously shown to decline in serially passaged MSCs [17, 46, 70]. Higher numbers of CD271^+^CD146^+^ cells were additionally found in fetal and paediatric bone marrow compared to adults [48]; however, our data revealed no significant differences in CD146 expression between the young and old donor groups, most likely because the present cohort did not include any paediatric donors. In relation to CD106, the literature pertaining to *in vitro* aged MSCs is controversial and indicates either its decline [70, 71], increase [32], or random oscillations [17] with passaging in standard culture conditions. Similar to CD146, we found no differences in the levels of CD106 expression on the surface of CD45^low^CD271^+^ BM cells in relation to donor age.

Altogether, our data reveal minimal transcriptional and phenotypic changes in BM CD45^low^CD271^+^ cells in old donors compared to young donors. An age-related decline in CD45^low^CD271^+^ BM cells was observed but not to the same degree as for CFU-Fs. Using the CFU-F assay and the same cohort of donors, we found lower proliferative ability of MSCs in older donors which was made worse with the addition of serum from old donors. Finally, large donor variation was observed in all age groups confirming that a person's biological age in terms of their BM MSC status is likely not to be the same as their chronological age.

Lower-than-expected differences in *in vivo* BM MSCs are in contrast to what has been described for long-term cultured, *in vitro* aged MSCs. This can be due to an enforced proliferation and rapid replicative aging in cultured MSCs, which do not normally occur *in vivo* where MSCs are normally quiescent. Even prior to their first passage in culture, MSCs normally undergo at least 10 population doublings [5, 7, 20], and further passaging to 20 population doublings has been estimated to “age” them by further 50 years, based on the telomere loss measurements [5]. Given that MSCs may in fact be long living and mostly quiescent *in vivo* [72], age-related changes in these cells may be more subtle and related to their metabolism or stress resistance, which would be interesting to investigate in the future. Additionally, recent studies have indicated that only a subpopulation of CD45^low^CD271^+^ BM cells may in fact be true self-renewing MSCs [30, 73], and the investigations of gene expression in these subpopulations are awaiting further investigation.

## 5. Conclusions

In summary, our data reveal a measurable reduction in MSC colony number and integrated density, as well as CD45^low^CD271^+^ cell number in older donors, confirming an age-related decline in number and proliferative capacity of MSCs. However, their selected multilineage gene expression profiles showed no age-related differences. In relation to therapeutic applications with minimally manipulated uncultured BM MSCs, our data indicate that old donor MSCs may be inferior to young donor MSCs in regard to their proliferation ability but not so much in terms of their multipotential gene expression. Furthermore, a person's chronological age is not a reliable predictor of their MSC number or potency, which merits further research to develop new assays for MSC biological fitness prior to their use in therapy.

## Data Availability

The data used to support the findings of this study are available from the corresponding author upon request.

## Supplementary Materials

Supplementary Materials Supplementary Table 1: preselected transcripts and their TaqMan assays used for gene expression. Supplementary Table 2: median fold differences of genes and surface markers between MSCs (CD45lowCD271^+^) and HLCs (CD45highCD271^−^) in the young and old donor groups.
