# Supplementary material for: The Analysis of In Vivo Aging in Human Bone Marrow Mesenchymal Stromal Cells Using Colony-Forming Unit-Fibroblast Assay and the CD45lowCD271+ Phenotype
Source: Stem Cells Int. 2019 Aug 1;2019:5197983. doi: 10.1155/2019/5197983 (PMC6701348; doi:10.1155/2019/5197983)
Supplement: Supplementary Materials — Supplementary Table 1: preselected transcripts and their TaqMan assays used for gene expression. Supplementary Table 2: median fold differences of genes and surface markers between MSCs (CD45lowCD271+) and HLCs (CD45highCD271−) in the young and old donor groups. [file 5197983.f1.docx]

| **Genes** | **Genes** | **Assay** |
| --- | --- | --- |
| TNFRSF11B/OPG | Osteoprotegerin | Hs00900360_m1 |
| SFRP1 | Secreted frizzled related protein 1 | Hs00610060_m1 |
| CXCL12 | C-X-C motif chemokine 12 | Hs00171022_m1 |
| FABP4 | Fatty acid binding protein 4 | Hs00609791_m1 |
| LepR | Leptin Receptor, encoding CD295 protein | Hs00174492_m1 |
| SPARC | Secreted protein acidic and rich in cysteine, Osteonectin | Hs00277762_m1 |
| TNFSF11/RANKL | Receptor activator of nuclear factor kappa-B Ligand | Hs01092186_m1 |
| PPAR-ɣ | Peroxisome proliferator activated receptor - gamma | Hs01115513_m1 |
| GJA1 | Gap Junction alpha 1, encoding Cx43 protein | Hs00748445_s1 |
| RUNX2 | Runt related transcription factor 2 | Hs00231692_m1 |
| HPRT | Hypoxanthine phosphoribosyl transferase (housekeeping) | Hs99999909_m1 |

**Supplementary table 1**

| **MSC v/s HLC** | **Young donors** | | **Old donors** | |
| --- | --- | --- | --- | --- |
|  | **Fold difference** | **p value** | **Fold difference** | **p value** |
| **OPG** | ND in HLCs | NA | ND in HLCs | NA |
| **SFRP-1** | ND in HLCs | NA | ND in HLCs | NA |
| **CXCL12** | 63428 | 0.0002 | 56182.2 | <0.0001 |
| **FABP4** | 269.2 | 0.0012 | 122.1 | <0.0001 |
| **LepR** | 245.3 | 0.0002 | 2050 | 0.0002 |
| **SPARC** | 139.50 | 0.0002 | 557 | <0.0001 |
| **RANKL** | 103.8 | 0.0003 | 24.2 | 0.0047 |
| **PPAR-g** | 55.2 | 0.0019 | 154.6 | <0.0001 |
| **GJA1** | 21.3 | 0.0047 | 11.3 | 0.0005 |
| **RUNX2** | 2.5 | 0.0541 | 3.2 | 0.0002 |
|  |  |  |  |  |
| **CD146** | 30.5 | 0.0115 | 28.9 | <0.0001 |
| **CD106** | 12 | 0.0029 | 10.3 | <0.0001 |
| **CD295** | 8.1 | 0.0005 | 9.3 | 0.0011 |
| **Cx43** | 0.5 | NS | 15.9 | 0.0079 |

ND: Not detected, NA: Not applicable, NS: Non significant

**Supplementary table 2**
